# Benign Multicystic Peritoneal Mesothelioma (BMPM) as a rare cause of abdominal pain: a case report and review of the literature

**DOI:** 10.1016/j.ijscr.2025.111448

**Published:** 2025-05-16

**Authors:** Hiba Ben Hassine, Midani Touati, Amal Bouchrika, Faiez Boughanmi, Ibtissem Korbi, Faouzi Noomen

**Affiliations:** Department of Visceral Surgery, Fattouma Bourguiba Hospital, Monastir, Tunisia

**Keywords:** Benign Multicystic Peritoneal Mesothelioma, Peritoneal neoplasm, Surgical management, Recurrence, Case report

## Abstract

**Introduction:**

Benign Multicystic Peritoneal Mesothelioma (BMPM) is a rare, benign cystic neoplasm arising from the peritoneum, predominantly affecting women of reproductive age. It is characterized by a slow progression and a high recurrence rate post-surgical resection. Due to its non-specific clinical presentation, BMPM remains a diagnostic challenge.

**Case presentation:**

We report a case of a 38-year-old female with a history of left ovarian cystectomy who presented with acute, stinging abdominal pain. Imaging studies, including ultrasound, contrast-enhanced computed tomography, and magnetic resonance imaging, revealed a large multicystic lesion in the left flank. Complete surgical excision was performed. Histopathology confirmed the diagnosis of BMPM.

**Discussion:**

Benign multicystic peritoneal mesothelioma (BMPM) is a rare, often asymptomatic cystic lesion predominantly affecting women of reproductive age, with unclear etiology possibly linked to hormones or prior inflammation. Imaging shows multiloculated cystic masses, and diagnosis is confirmed histologically and immunohistochemically. Surgical resection is the main treatment due to high recurrence, with close follow-up recommended.

**Conclusion:**

BMPM is a rare peritoneal neoplasm with a challenging diagnosis due to its non-specific symptoms and imaging characteristics. Complete surgical resection remains the mainstay of treatment, although close follow-up is necessary due to its high recurrence potential.

## Highlights

• BMPM is a rare, benign cystic tumor of the peritoneum, predominantly seen in women of reproductive age.
• The clinical presentation is non-specific making diagnosis difficult.
• Imaging (US, CT, MRI) typically shows a large, multiloculated, thin-walled cystic mass
• Histopathology and immunohistochemistry are essential for definitive diagnosis,
• Surgical resection remains the mainstay of treatment,

## 1 Introduction

Benign Multicystic Peritoneal Mesothelioma (BMPM) is a highly uncommon, non-malignant cystic tumor originating from the peritoneal lining [1], predominantly affecting women [2]. It typically demonstrates a slow-growing nature and is notable for a high recurrence rate following surgical removal [3]. Initially identified by Mennemeyer and Smith in 1979, the global literature included fewer than 200 documented cases as of 2020 [4]. Clinical presentation is often vague, including symptoms such as abdominal or pelvic discomfort and the presence of a mass. Currently, there are no standardized, evidence-based protocols for management [5]. In this report, we present a case involving a 38-year-old woman diagnosed with BMPM who underwent complete surgical excision. Histopathological analysis confirmed the diagnosis. This case is documented by the 2023 SCARE criteria [6].

## 2 Case presentation

**Fig. 1:**
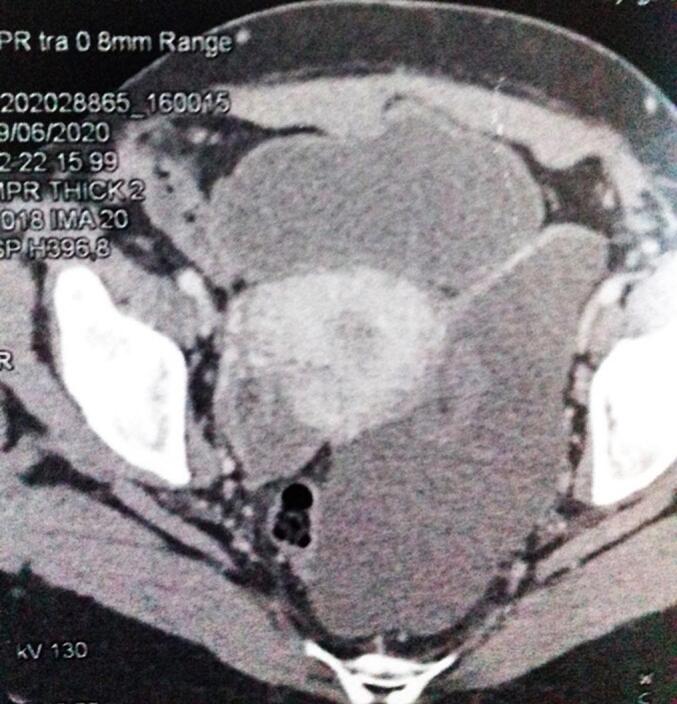
Abdominal CT scan of BMPM: polylobate hypodense formation with multiple thin internal septa which are enriched after contrast.

**Fig. 2:**
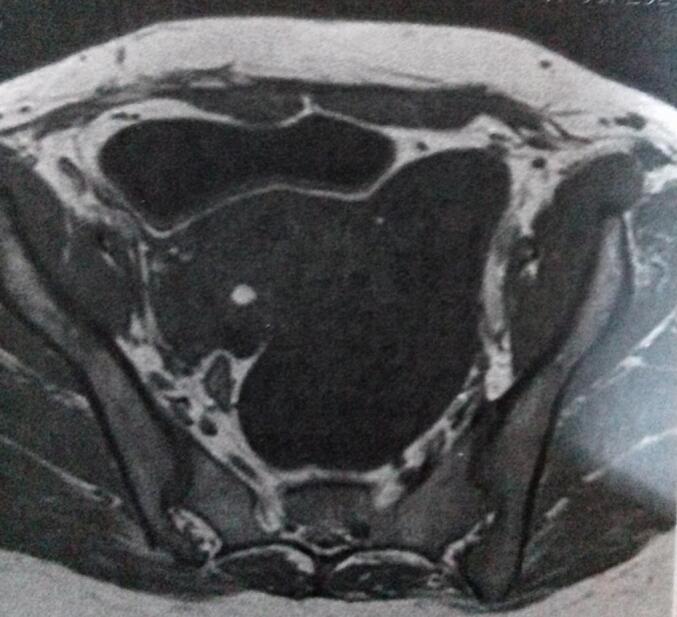
MRI demonstrating cystic lesion posterior to the urinary bladder in the left para-colic gutter.

**Fig. 3:**
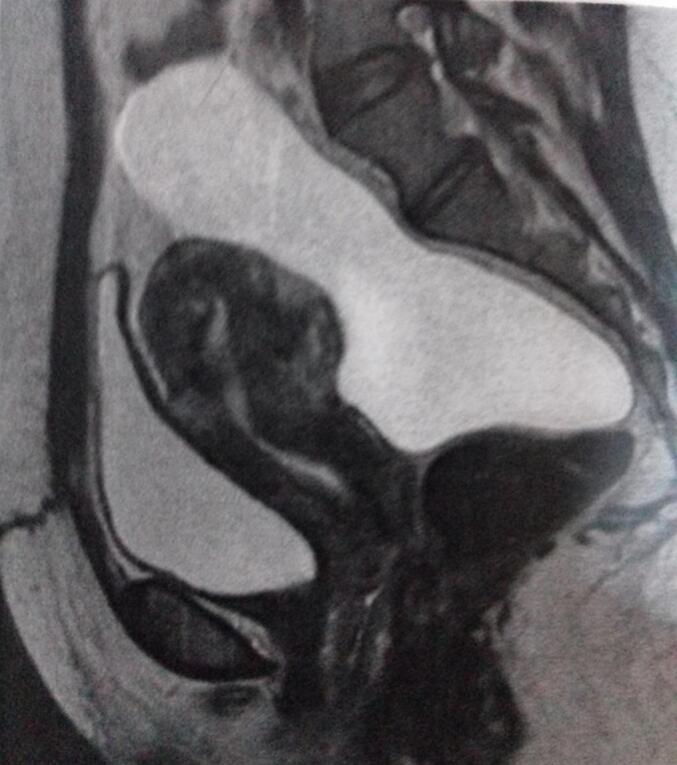
MRI demonstrating cystic lesion with multiple thin internal septa.

A 38-year-old woman, with a medical history of left ovarian cystectomy performed five years prior, presented with intermittent abdominal discomfort persisting for the past three years. She recently experienced a sudden, sharp pain localized to the left side of her abdomen. On physical examination, no abnormalities were noted. Laboratory blood tests and tumor markers were within normal limits. An abdominal ultrasound revealed a multicystic mass measuring approximately 15 cm in diameter on the left flank. Further evaluation with contrast-enhanced computed tomography (CT) identified a hypodense, multilobulated lesion containing multiple thin internal septations that enhanced with contrast (dimensions: 150 × 95 mm) (Fig. 1). Magnetic resonance imaging (MRI) supported these findings (Fig. 2, Fig. 3).

**Fig. 4:**
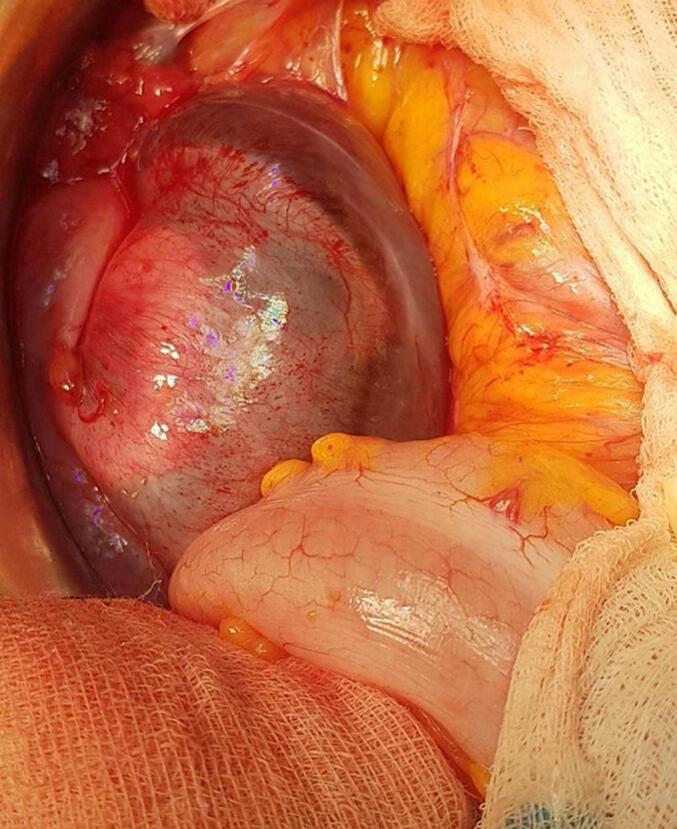
Intraoperative images showing multicystic grapelike MASS.

**Fig. 5:**
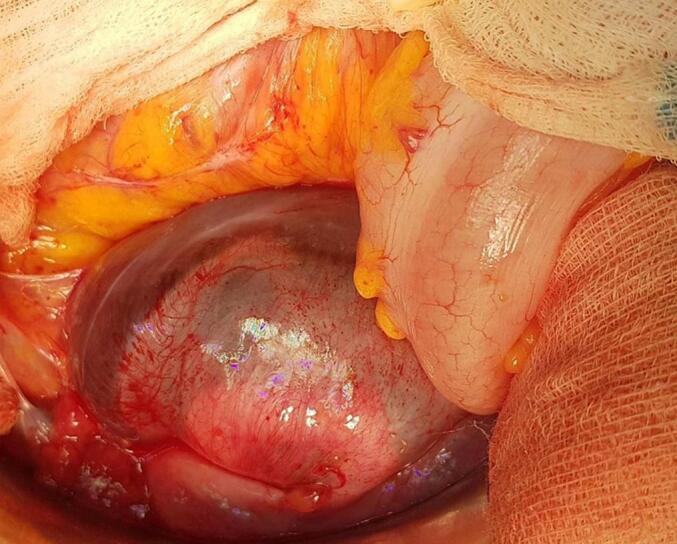
Intraoperative image showing multicystic grapelike mass with a weak adherence with the descending colon and rectum.

Following a multidisciplinary team discussion, exploratory laparotomy was performed. Intraoperative findings included numerous cystic lesions arising from the peritoneum, with some involving the omentum and showing minimal adhesions to the descending colon and rectum (Fig. 4, Fig. 5). A complete surgical excision was carried out. The postoperative recovery was smooth, and the patient was discharged on the fourth postoperative day without any complications. Histopathological analysis revealed multiple cystic structures lined by a single layer of mesothelial cells, with fibrous walls containing adipose tissue. The cysts were filled with a clear serous fluid. The final diagnosis was benign multicystic peritoneal mesothelioma. At the ten-month mark post-surgery, the patient remains recurrence-free.

## 3 Discussion

Peritoneal mesothelioma is a rare condition that arises from mesothelial cells lining the body's serous cavities. Its incidence is estimated at approximately 1 per 1,000,000 individuals [4]. The initial description was made by Mennemeyer and Smith in 1979 [4]. This disease predominantly affects women of childbearing age, with a female-to-male ratio of 4–5:1. Cases occurring in women over the age of 30 are exceptionally rare [9]. The exact origin of benign multicystic peritoneal mesothelioma (BMPM) remains debated. It has been linked to conditions such as pelvic inflammatory disease, endometriosis, uterine fibroids, and prior abdominal or pelvic surgeries like hysterectomy or tubal ligation [10]. Nonetheless, reports of BMPM in patients without any history of surgery or inflammation have led some researchers to support a neoplastic origin. Over the years, a variety of terms have been used to describe this entity, such as multilocular peritoneal inclusion cyst, cystic mesothelioma, peritoneal mesothelial cyst, inflammatory peritoneal cyst, and postoperative peritoneal cyst—highlighting the lack of consensus on its nature and behavior [9]. Another theory proposes a hormonal influence, with the tumor's development and progression possibly related to hormonal sensitivity, supported by the high frequency of BMPM in reproductive-aged women [13]. Unlike pleural mesothelioma, BMPM has not been linked to asbestos exposure [12]. BMPM is most frequently diagnosed in women between the ages of 20 and 40; however, cases outside this age range have been reported, but are less common. [5,10]. Most patients are asymptomatic until the lesion becomes large enough to compress surrounding structures. Common initial symptoms include vague abdominal discomfort, tenderness, a palpable mass, or unintentional weight loss. Due to the tumor's preference for the pelvic peritoneum, it may involve structures such as the rectum, bladder, or uterus, leading to symptoms like dyspareunia, urinary disturbances, or bowel obstruction [14]. Ultrasound imaging usually reveals multiloculated, anechoic cystic structures. A characteristic “spider-in-web” appearance may also be noted [14]. CT scanning is useful for defining the lesion's extent and location; typically, it shows a low-attenuation, thin-walled, multicystic mass [15]. MRI is considered the most informative imaging modality, with cystic areas appearing hyperintense on T2-weighted images and contrast enhancement often seen in the septa following gadolinium administration [8]. The differential diagnosis of BMPM is broad, including both benign and malignant conditions. Benign mimickers include retroperitoneal cystic lymphangioma, endometriosis, and cystic adenomatoid tumors. Malignant lesions that may resemble BMPM include malignant mesothelioma, peritoneal serous tumors, and ovarian clear cell carcinoma. On histology, BMPM typically displays multiple cystic spaces lined by a single layer of flattened or cuboidal mesothelial cells. These cysts vary in size and are filled with a pale, proteinaceous fluid. There are usually no signs of nuclear atypia. Immunohistochemically, the mesothelial lining is positive for markers such as epithelial membrane antigen (EMA), calretinin, CA-125, Wilms' tumor protein (WT1), vimentin, D2–40, and cytokeratins. Management of BMPM remains a subject of debate. Treatment options range from observation to aggressive surgical resection. Some experts advocate for conservative monitoring in asymptomatic individuals, although there is currently no consensus on optimal follow-up schedules or imaging intervals [7]. Surgical excision remains the most widely accepted approach, primarily due to the high recurrence rate, which ranges between 50 % and 60 % postoperatively. Although laparotomy was selected in our case due to the suspected extent of adhesions and mass effect, a laparoscopic approach might be preferable in selected cases to reduce peritoneal trauma, which has been postulated as a possible trigger for recurrence. Hormonal influence, particularly involving estrogen, has been implicated in the pathogenesis of BMPM. Some authors have reported responses to hormonal therapies, including tamoxifen and gonadotropin-releasing hormone analogues. Alternative therapies, including hormonal treatments, sclerotherapy, and potassium-titanyl phosphate (KTP) laser vaporization, have been explored. While KTP laser therapy has shown good tumor penetration, its long-term efficacy remains uncertain [9]. Given the high likelihood of recurrence, close postoperative surveillance is recommended. Strategies to reduce recurrence include complete cytoreductive surgery and, in selected centers, hyperthermic intraperitoneal chemotherapy (HIPEC). However, evidence supporting their efficacy in BMPM is limited. Some authors propose conducting CT scans every three months during the first year, followed by annual imaging for up to five years [16].

## 4 Conclusion

MCPM is an uncommon benign tumor that lacks specific symptoms, clinical features, or characteristic imaging findings. As a result, the diagnosis often remains uncertain until histopathological confirmation is obtained. This case aims to raise awareness about this rare condition. Once BMPM is definitively diagnosed, surgical intervention should be pursued with the objective of removing both visible and microscopic disease.

## Author contribution

Hiba Ben Hassine, participated in the treatment of the patients and writing the manuscript.

Hiba Ben Hassine, Amal bouchrika, Midani Touati, Faiez Boughanmi, Ibtissem Korbi, Faouzi Noomen: validation of the manuscript.

All the authors approved the manuscript.

## Consent

Written informed consent was obtained from the patient for publication of this case report and accompanying images. A copy of the written consent is available for review by the Editor-in-Chief of this journal on request.

## Consent for publication

The patient gave written consent for their personal or clinical details along with any identifying images to be published.

All participants gave written consent for their personal or clinical details along with any identifying images to be published in this study.

## Ethical approval

The study was approved by Ethics Committee of Hospital Fattouma Bourguiba Monastir.

## Guarantor

Hiba Ben Hassine.

## Research registration number

Not applicable.

## Funding

This research received no specific grant from the public, commercial, or not-for-profit sectors.

## Conflict of interest statement

No conflict of interest to disclose. The authors declare no competing interests. The study did not receive any sources of support or funding.
